# Nondestructive Evaluation of Carbon Fiber Reinforced Polymer Composites Using Reflective Terahertz Imaging

**DOI:** 10.3390/s16060875

**Published:** 2016-06-14

**Authors:** Jin Zhang, Wei Li, Hong-Liang Cui, Changcheng Shi, Xiaohui Han, Yuting Ma, Jiandong Chen, Tianying Chang, Dongshan Wei, Yumin Zhang, Yufeng Zhou

**Affiliations:** 1 College of Instrumentation and Electrical Engineering, Jilin University, Changchun 130061, China; zhangjin0109@jlu.edu.cn (J.Z.); hanxiaoh52@163.com (X.H.); mayuting1992@foxmail.com (Y.M.); chenjd13@mails.jlu.edu.cn (J.C.); changtianying@hotmail.com (T.C.); 2 Research Center for Terahertz Technology, Key Laboratory of Multi-scale Manufacturing Technology, Chongqing Institute of Green and Intelligent Technology, Chinese Academy of Sciences, Chongqing 400714, China; liwei@cigit.ac.cn (W.L.); dswei@cigit.ac.cn (D.W.); 3 National Key Laboratory of Science and Technology on Advanced Composites in Special Environments, Harbin Institute of Technology, Harbin 150080, China; zhym@hit.edu.cn (Y.Z.); zhouyf@hit.edu.cn (Y.Z.)

**Keywords:** carbon fiber reinforced polymer composites, fire-retardant coatings, nondestructive evaluation, coating debonding, terahertz time-domain spectroscopy imaging

## Abstract

Terahertz (THz) time-domain spectroscopy (TDS) imaging is considered a nondestructive evaluation method for composite materials used for examining various defects of carbon fiber reinforced polymer (CFRP) composites and fire-retardant coatings in the reflective imaging modality. We demonstrate that hidden defects simulated by Teflon artificial inserts are imaged clearly in the perpendicular polarization mode. The THz TDS technique is also used to measure the thickness of thin fire-retardant coatings on CFRP composites with a typical accuracy of about 10 micrometers. In addition, coating debonding is successfully imaged based on the time-delay difference of the time-domain waveforms between closely adhered and debonded sample locations.

## 1. Introduction

Carbon fiber reinforced polymer (CFRP) composites are widely used in concrete structure reinforcement, the automotive industry, and aerospace engineering due to their low cost, light weight and high strength [1,2]. However, CFRP composites can be damaged by various factors during the manufacturing processes and in deployment. In addition, fire-retardant coatings are usually brushed or sprayed on the surface of CFRP composites to ensure their structural reinforcement effect when exposed to fire [3]. If the coatings are too thin, they cannot play their fire prevention role. On the other hand, if the coatings are too thick, not only are the coating materials wasted, but also the bonding strength of the coatings decreases as the stress increases. In addition, if the fire-retardant coatings are debonded, they cannot be functional in a fire. Based on the above reasons, advanced technologies are required to detect the defects of CFRP composites and fire-retardant coatings.

The common nondestructive evaluation (NDE) methods are the ultrasonic [4], eddy current [5], X-ray [6] and so on, which have their respective strengths and weaknesses. For example, close contact with samples is required for ultrasonic technologies, a conductive substrate and contact with samples are required for eddy current technologies, and X-ray methods use harmful radiation. Recently, terahertz (THz) time-domain spectroscopy (TDS) imaging technology has received increasing attention as a non-destructive, non-ionizing, and non-contact NDE method [7,8] in industrial systems.

To date, only a limited number of studies on the THz NDE of CFRP composites exist [9,10], especially for thickness and defect inspection of fire-retardant coatings on the surface of CFRP. In this study, using a reflective THz TDS system we examine CFRP composites with various defects, such as Teflon artificial inserts, uneven coating thickness and coating debonding to demonstrate its NDE capability. Hidden Teflon defects are detected successfully at 0.075 THz, where the difference of refractive index between the epoxy resin and Teflon is the largest. Moreover, we not only measure the thickness of fire-retardant coatings, but also use THz imaging technology to image uneven coating regions, from which we could easily see the distribution of the coating thickness. Finally, the coating debonding defects are detected satisfactorily, with a minimum detectable thickness of debonding of approximately 150 µm.

The remainder of this paper is organized as follows: first, we briefly introduce the THz TDS imaging system and the samples used in our experiments in Section 2. Section 3 describes the related theories of refractive index measurement and the imaging algorithm. The experimental results and analyses are given in Section 4. Finally, concluding remarks and a summary are presented in the last section.

## 2. THz Imaging Systems and Samples

**Figure 1:**
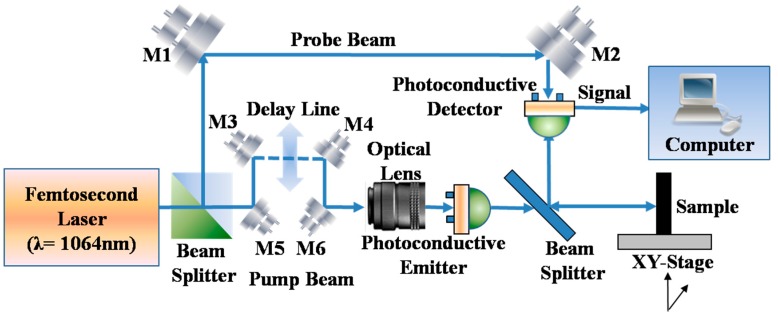
Schematic diagram of the THz TDS system (M1–M8: Mirrors).

In this study, we employ a THz TDS system T-Ray 5000 [11] from Advanced Photonix, Inc. (API, Ann Arbor, MI, USA). Figure 1 shows a schematic diagram of the THz TDS system in the reflection mode. The main parameters of the system are as follows: a time domain range of 0 to 80 ps with 0.1 ps resolution, a spectral range of 0 to 3.5 THz with a spectral resolution of 12.5 GHz. The signal to noise ratio in the reflection mode is better than 40 dB at the low-frequency end, and the effective measurement range is about 0–1.8 THz. The details about the measurement methods and conditions of signal to noise ratio are covered in Section 1 of the Supplementary Information. The raster scan imaging area of the xy-stage is up to 30 × 30 cm, and the minimum step size is 0.1 mm both horizontally and vertically. Furthermore, the imaging rate of the system is relatively high, and a 100 × 100 pixel image can be acquired in as little as 16 s. In this paper, unless otherwise noticed, all the imaging results are obtained in reflection mode.

The samples used in this study are classified into several categories. Firstly, unidirectional CFRP samples are prepared for a THz spectroscopic study, which are respectively 1-, 2-, 3- and 4-ply, with corresponding thicknesses of 0.14, 0.31, 0.42 and 0.55 mm. In addition, the orientations of the carbon fibers in our samples are all the same. The CFRP sample consists of carbon fiber and epoxy resin. The diameter of a single carbon fiber is about 7 µm, and the distance between the single carbon fibers is smaller than 1 µm. Secondly, a CFRP sample with Teflon artificial insert is fabricated to simulate a foreign inclusion or an inter-ply delaminational defect. Thirdly, the fire-retardant coatings with different thicknesses are brushed on the surface of CFRP composites. Finally, a CFRP sample with thin type debond coating is fabricated.

## 3. Theoretical Background

### 3.1. Refractive Index

When THz wave is normally incident, one of the methods to calculate the refractive index in the reflection mode is based on the optical path length difference between the front and back reflections in the time domain [12], which is defined as follows:

(1) $$n=\frac{ct}{2d}$$

where t is the transmission time of the sample, d is the sample thickness, and c is the THz wave propagation speed in the air.

### 3.2. Imaging Algorithm

During the imaging process [13], the time-domain waveforms are measured for every pixel. Through a Fourier transform, the corresponding frequency-domain waveforms are obtained. For time-domain imaging, an image can be built up pixel by pixel by such features of the waveform as the maximum peak, minimum peak, peak-to-peak, time-of-flight, and so on. For frequency-domain imaging, one could use such information as amplitude, power, power spectral density, phase, refractive index and absorption coefficient at certain frequency or integrated over a selected frequency interval. For different samples, the information which can produce the best imaging effect is different. In our experiments, the imaging algorithms are not the same for each sample. For the CFRP sample with a Teflon artificial insert, the hidden defects are detected successfully through the reflected power imaging at 0.075 THz. Uneven coating thickness imaging is built up depending on the time delay imaging between coating surface and substrate reflection. The difference of the reflection time-domain waveforms between closely adhered and debonding locations is used to detect the debonded coating.

## 4. Results and Discussion

### 4.1. THz Spectroscopic Properties of CFRP

**Figure 2:**
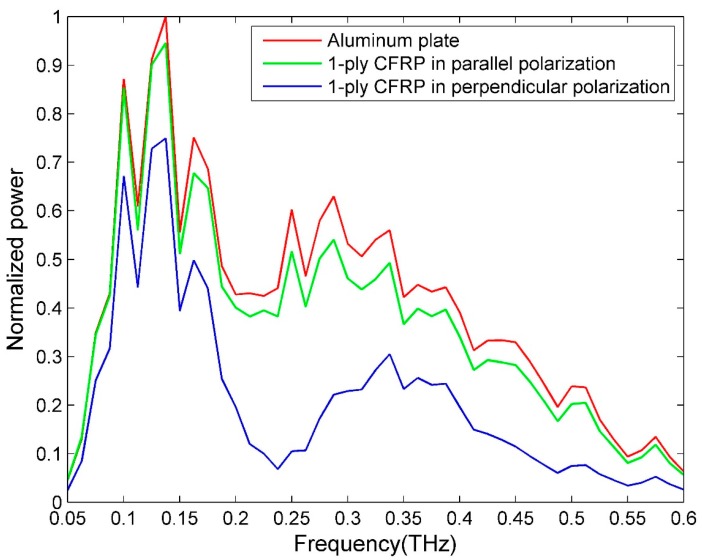
The reflectivity comparison of an aluminum plate and a 1-ply CFRP.

Because CFRP is a type of conductive composite, it exhibits a high THz reflectivity. Furthermore, the reflectivity is highly dependent on the polarization of the incoming THz radiation and fiber orientation due to the anisotropy of CFRP’s conductivity. Figure 2 shows the reflectivity comparison between an aluminum plate (the reference in the reflection mode) and a 1-ply CFRP. As can be seen from Figure 2, when the fiber direction is parallel to the electric field direction (parallel polarization), the reflected THz power is close to that of the aluminum plate, and much higher than the case with perpendicular polarization. In the perpendicular polarization mode, the THz transmitted power is the highest for unidirectional CFRP. Because the best imaging frequency for the CFRP sample with a Teflon artificial insert defect is 0.075 THz, we pay special attention to the transmitted power comparison at 0.075 THz. The transmitted power at 0.075 THz are 17.14%, 13.55%, 21.37% (the existence of Fabry-Pérot interference) and 8.16%, relative to air, which serves as the reference in the through-transmission mode, for 1-ply, 2-ply, 3-ply and 4-ply, respectively. The significant peaks for the reference signal are from the system, and the details are presented in Section 2 of the Supplementary Information.

### 4.2. Teflon Artificial Insert

**Figure 3:**
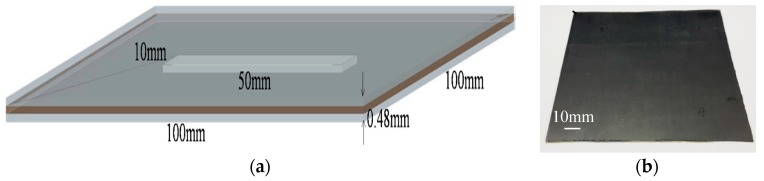
A CFRP sample with a Teflon artificial insert: (**a**) The three-dimensional rendering; (**b**) Front side optical image.

**Figure 4:**
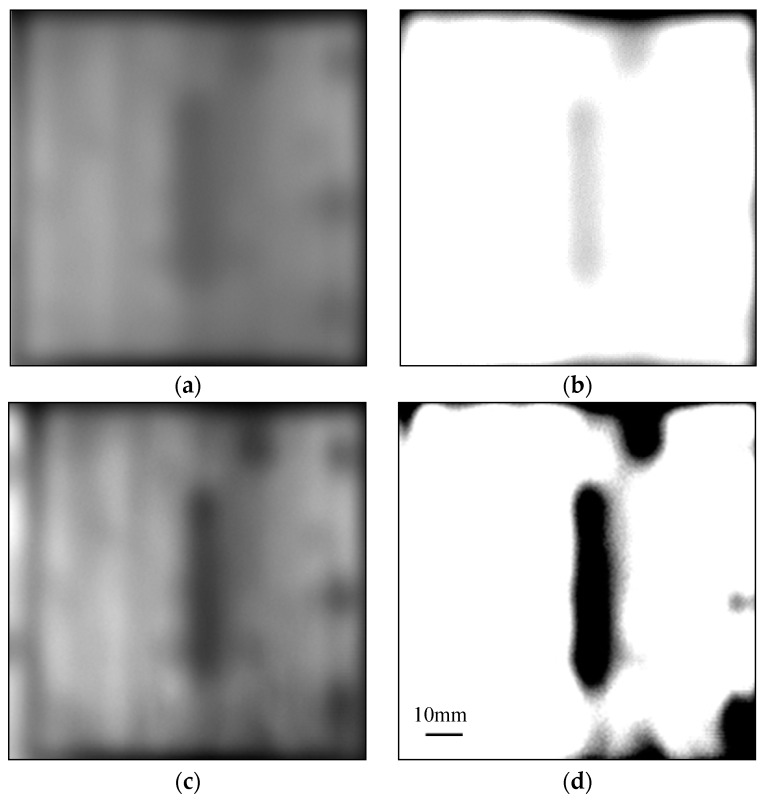
The reflective imaging results of a Teflon artificial insert defect in the perpendicular polarization mode: (**a**) THz power image from 0.053–0.088 THz; (**b**) The result after an image enhancement process of (**a**); (**c**) THz power image at 0.075 THz; (**d**) The result after an image enhancement process of (**c**).

**Figure 5:**
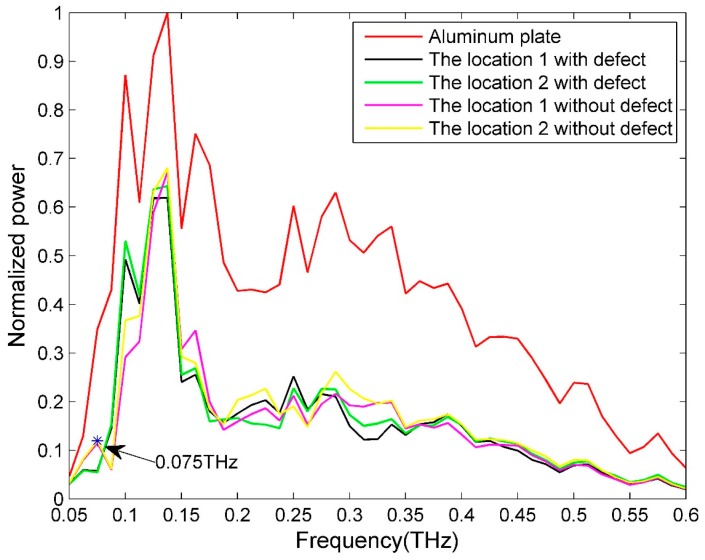
The typical reflective power spectrum measured on an aluminum plate, CFRP with defect and without defect.

**Figure 6:**
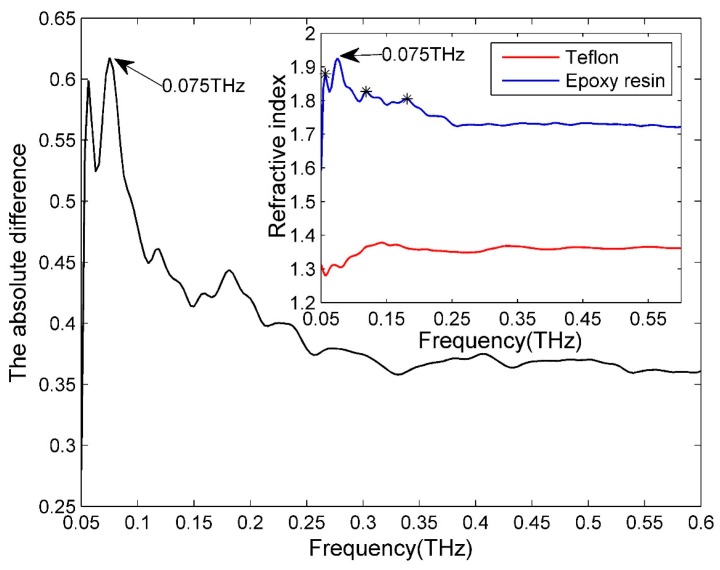
The absolute difference of the refractive index between epoxy resin and Teflon.

Figure 3a shows the three-dimensional (3D) rendering of a CFRP sample with a Teflon artificial insert. The Teflon sheet (50 × 10 × 0.2 mm^3^) is inserted between two single-ply unidirectional CFRP sheets (100 × 100 × 0.14 mm^3^) through a hot-press process. Hence, the material is Teflon between two CFRP sheets in the defect region, whereas it is epoxy resin elsewhere. From Figure 3b, we can see that the surface of the sample is flat, without apparent features. The reflective imaging results of this sample are shown in Figure 4. Because the incident THz waves are almost fully reflected by the CFRP for parallel polarization whereas THz radiation can penetrate deeper into the CFRP composite in the case of perpendicular polarization, the angle between the THz electric field direction and the fiber direction is fixed at 90 degrees in this test. Figure 4a shows pixels that represent the sums of reflected powers from 0.053 to 0.088 THz, and the artificial defect can barely be seen. Figure 4c corresponds to the reflected power distribution at 0.075 THz, and we can clearly see the hidden defect. The experimental results can be explained as follows. Figure 5 is the typical reflective power spectrum measured on an aluminum plate, CFRP with defect and without defect. From Figure 5, we can see that the normalized reflective power at 0.075 THz for two locations of CFRP with defect is similar, and these values of CFRP without defects are also comparable. However, the difference between CFRP with defect and without defect is significant. Such an obvious feature is suitable for detecting and imaging the defects. The stability of the reflected power signal at 0.075 THz is verified in Section 1 of the Supplementary Information. In addition, as can be seen from Figure 6, the maximum absolute difference of refractive index between epoxy resin and Teflon is also at 0.075 THz. The details about the refractive index of epoxy resin and Teflon are shown in Section 3 of the Supplementary Information. The reflected powers of the defect region differ the most from the rest of the sample at 0.075 THz according to Fresnel’s Law of Reflection [14]. After a linear-contrast-enhancement image processing [15] for Figure 4a,c, the defect becomes much clearer, as shown in Figure 4b,d. The darker areas at the top and bottom right corners (clearly seen in Figure 4d) may be caused by the inhomogeneity of the epoxy resin adhesive between two single-ply unidirectional CFRP sheets, which are more prevalent around the edges of the sample. They are probably due to press-pressure induced uneven spill of the epoxy toward the edges of the sample during fabrication. The four small squares at the right edge (better seen in Figure 4c) might be the impression of clamp holders from when the sample was made. The carbon fibers became twisted due to the pressure of clamp holders, which cannot be seen in the naked eye. The best imaging effect is performed in frequency domain at 0.075 THz, and the scanning step size in this experiment is 0.5 mm, so the lateral resolution is about 6 mm, which is described in details in Section 4 of the Supplementary Information.

### 4.3. Uneven Coating Thickness

**Figure 7:**
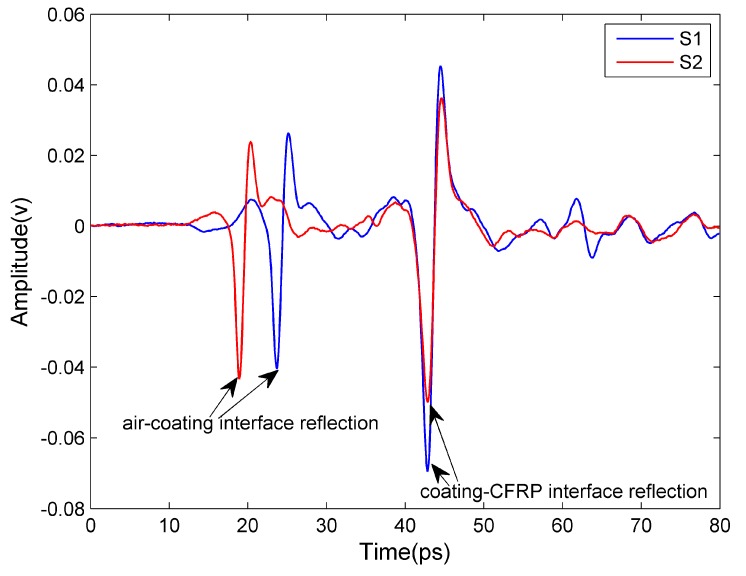
The time-domain waveforms reflected from CFRP sample with ultra-thin fire-retardant coatings.

The results of measurements of fire-retardant coating thickness by THz imaging are reported in this subsection. Fire-retardant coatings can be divided into three categories: ultra-thin, thin and thick type, which have different compositions. The range of thickness is respectively less than 3 mm for ultra-thin type, 3–7 mm for thin type, and 8–50 mm for thick type. In this study, we only measured ultra-thin and thin type fire-retardant coatings. For thick type fire-retardant coatings, the time delay between the coating surface and substrate reflection is beyond the time domain range of our system (80 ps). Figure 7 shows the time-domain waveforms reflected from two CFRP samples with ultra-thin fire-retardant coatings of different thicknesses (about 1.62 mm for sample S1 and 2.02 mm for sample S2). The first main peak is caused by the reflection at the interface between air and the coating, whereas the second corresponds to the interface between the coating and the CFRP substrate. The time difference between the two main reflection peaks is proportional to the coating thickness.

**Figure 8:**
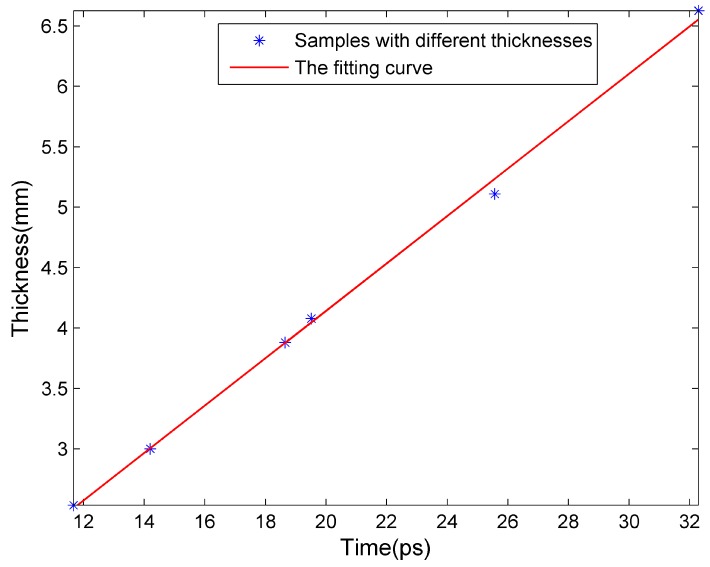
The linear dependence of THz propagation time on coating thickness on thin fire-retardant coatings on CFRP.

**Figure 9:**
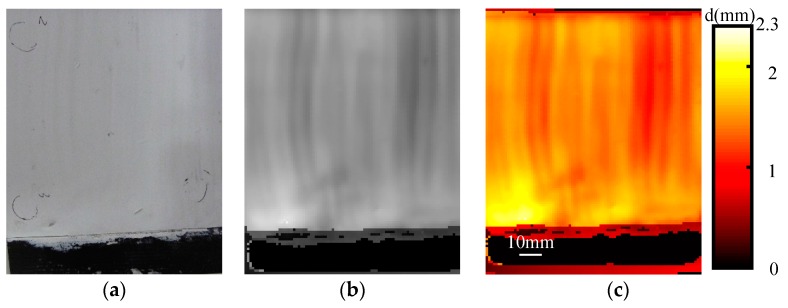
The reflective imaging results of uneven coating thickness: (**a**) Front side optical image; (**b**) THz imaging using the propagation time in the coating; (**c**) The result after a pseudo-color conversion process of (**b**).

The CFRP samples with thin fire-retardant coatings of several known thicknesses were measured, which are shown as blue points in Figure 8. The red curve in Figure 8 represents a linear fitting of the data, and the goodness of fit statistics is 99.79%. Hence, for the samples with thin fire-retardant coatings of unknown thickness, we can calculate the coating thickness through the measured propagation time and the linear fitting relationship. Figure 9 shows the imaging results of uneven coating thickness. The black region in Figure 9a is the CFRP substrate without fire-retardant coating, whereas the white region is the fire-retardant coating on the surface of CFRP. Figure 9b is built up pixel by pixel by the propagation time of THz wave in the coating. The higher image gray value corresponds to thicker coating. After a pseudo-color conversion process, the coating thickness difference becomes more obvious, as seen in Figure 9c. The imaging is performed in time domain, and the scanning step size in this experiment is 1 mm, so the lateral resolution is nearly between 1 and 2 mm, which is described in details in Section 4 of the Supplementary Information.

The measurement range of the coating thickness for our system can be calculated as follows:

(2) $$\frac{ct_{\min}}{2n}\leq d_{z}\leq \frac{ct_{\max}}{2n}$$

where *c* is the THz wave propagation speed in the air (3 × 10^8^ m/s), and *n* is the refractive index of the coating (after calculation using Equation (1), 1.76 for ultra-thin type and 1.46 for thin type). *t*_min_ and *t*_max_ are respectively the minimum and maximum time differences between the coating surface and the substrate reflection. In order to clearly distinguish the waveforms returned from the coating surface and the CFRP substrate, *t*_min_ cannot be less than half of the THz pulse width (about 4 ps for a full THz pulse width in our case); and *t*_max_ cannot be more than the time window (80 ps in our case). Hence, *t*_min_ and *t*_max_ are set to 2 ps and 80 ps, respectively. According to Equation (2), we estimate that our measurement range of coating thickness is 0.17–6.82 mm and 0.21–8.22 mm for ultra-thin and thin type, respectively, meeting satisfactorily most practical detection requirements of fire-retardant coatings.

The measurement resolution of coating thickness for our system can be obtained through the following equation:

(3) $$\Delta_{d_{z}}=\frac{c}{2n}*\Delta_{t}$$

where Δ*_t_* is the resolution of time delay in our system (0.1 ps). According to Equation (3), we estimate that the measurement resolution of coating thicknesses is 8.52 µm and 10.27 µm for ultra-thin and thin type, respectively.

### 4.4. Coating Debonding

**Figure 10:**
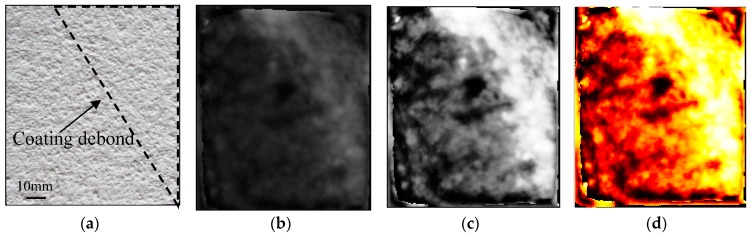
The reflective imaging results of coating debond: (**a**) Front side optical image; (**b**) THz imaging result; (**c**) The result after an image enhancement process for (**b**); (**d**) The result after a pseudo-color conversion process for (**c**).

**Figure 11:**
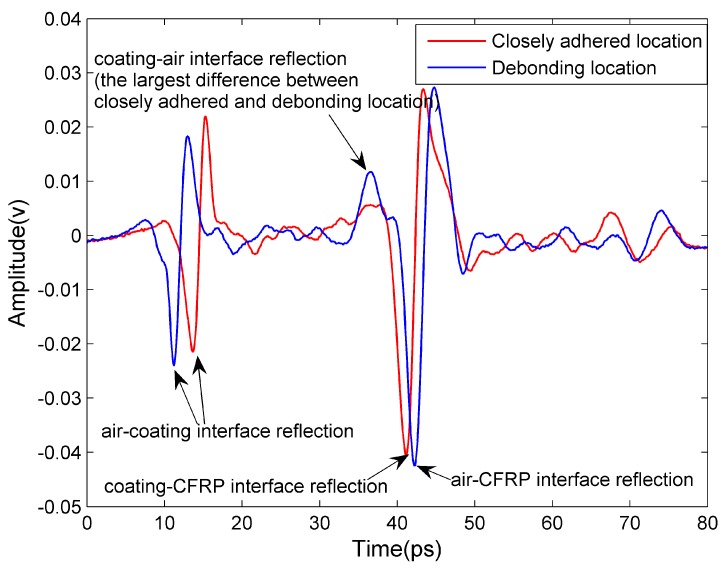
The time-domain waveforms reflected from CFRP sample with thin fire-retardant coatings.

This subsection describes the results of detection of thin fire-retardant coating debonding. In the upper right corner of the sample, the coatings are deliberately debonded, which is approximately shown in the black dashed-line enclosed region of Figure 10a. The average coating thickness of this sample is 2.5 mm, and the range of thickness of the debonded area is approximately 150–700 µm. In reality, the common causes of debonding are uncleanliness and/or unevenness of the substrate surface, exposure to excessive moisture and high temperature/low temperature cycles, *etc*. In order to mimic the debonding, we brushed the fire-retardant coatings on the unclean CFRP substrate. After a few days, the coatings debonded gradually, resulting in a debonded zone with an air gap. The time-domain waveform reflected from closely adhered region of the CFRP sample is compared with that from the debonded region, as seen in Figure 11.

An interesting aspect of the measurement is the polarity of the reflected waveform for reflection measurements [16]. For closely adhered locations, the two main reflections are both from a low to high refractive index material (air to coating and coating to CFRP), so the polarities of the two reflection waveforms are the same (from negative to positive). For the debonding locations, on the other hand, the three main reflections are respectively from air to coating (a low to high refractive index material), coating to air (high to low) and air to CFRP (low to high), so the polarity of the second reflection (from positive to negative) is different from the first and third (from negative to positive). Moreover, the thickness of the air debonding layer is so small that the second and third waveforms are somewhat overlapping. Hence, as can be seen from Figure 11, the most obvious difference of the time-domain waveforms between closely adhered and debonding locations is the emergence of a positive peak for debonding locations before the reflection of the surface of CFRP (air to CFRP interface reflection). In this work, we use the value of the aforementioned positive peak to build up the THz image, with the debonding defect shown in Figure 10b. After a linear-contrast-enhancement processing [15], the defect becomes much clearer, as shown in Figure 10c. The lateral resolution in this subsection is the same as that in Section 4.3.

## 5. Conclusions

In conclusion, we have conducted a series of studies on defect detection of the CFRP composites and fire-retardant coatings using a reflective THz TDS imaging technique. Due to the strong reflectivity of CFRP and relatively low energy of THz TDS systems, THz TDS technology is only suitable for thin CFRP-dielectric sandwiched structures in the perpendicular polarization mode. However, the strong reflectivity of CFRP makes THz technology an excellent choice for the detection of thin coatings on them, such as fire-retardant coatings (and other dielectric coatings), including coating thickness and coating debonding. Although THz technology has achieved some enviable results for defect detection, THz imaging is also been confronted with many obstacles, such as low resolution and inadequate image processing algorithms which are not usually customized for THz imaging applications. The imaging resolution can be improved by improvement in the requisite hardware, but it will be achieved at a high cost. We will instead focus on the high-resolution reconstruction algorithms for terahertz imaging in the near term. In addition, THz images obtained through different features usually show different aspects of defect information, it is then reasonable to speculate that suitable image fusion approaches and attendant algorithms may be able to combine relevant information from two or more THz images into a single image, which may be more informative than any of the single-input THz images, akin to the image-fusion approach used in infrared and visible imaging, and hyper-spectral imaging employed in remote sensing applications [17,18]. Work along this direction is currently underway in our laboratory, and we expect to report on it in the near future.

## Supplementary Materials

The following are available online at http://www.mdpi.com/1424-8220/16/6/875/s1, Figure S1: Schematic diagram of the THz TDS system (M1–M8: Mirrors), Figure S2: The signal to noise ratio of the THz TDS system in both the through-transmission and reflection mode, Figure S3: The corresponding mean and standard deviation of the reference and noise signals in the through-transmission mode, Figure S4: The corresponding mean and standard deviation of the reference and noise signals in the reflection mode, Figure S5: Optical images of the standard resolution test pieces with rectangular holes of different widths on an aluminum plate: (a) 1–5 mm; (b) 6–9 mm, Figure S6: The reflective imaging results of standard resolution test pieces: (a) the peak-to-peak amplitude image in time domain; (b) THz power image at 1 THz; (c) THz power image at 0.5 THz; (d) THz power image at 0.1 THz; (e) THz power image at 0.075 THz for the rectangular holes of different widths from 1 to 5 mm; (f) THz power image at 0.075 THz for the rectangular holes of different widths from 6 to 9 mm.
