# Supplementary material for: Nondestructive Evaluation of Carbon Fiber Reinforced Polymer Composites Using Reflective Terahertz Imaging
Source: Sensors (Basel). 2016 Jun 14;16(6):875. doi: 10.3390/s16060875 (PMC4934301; doi:10.3390/s16060875)
Supplement: Supplementary file 1 [file sensors-16-00875-s001.pdf]

Supplementary Materials: Nondestructive Evaluation of Carbon Fiber Reinforced Polymer Composites Using Reflective Terahertz Imaging

Jin Zhang, Wei Li, Hong-Liang Cui, Changcheng Shi, Xiaohui Han, Yuting Ma, Jiandong Chen, Tianying Chang, Dongshan Wei, Yumin Zhang and Yufeng Zhou

1. The Characterization of Signal to Noise Ratio and Spectral Stability for the THz TDS System

Systematic experiments are carried out to characterize the stability of the spectrum in both the through-transmission and the reflection mode. Figure S1 shows a schematic diagram of the THz TDS system in both modes. In the through-transmission mode, we measure the reference signal when the THz wave transmits through the air, and obtain the background noise signal when the THz transmitter is fully blocked. In the reflection mode, the reference signal is measured when a smooth aluminum plate is placed at the focal point, and the background noise signal is acquired when nothing is placed in front of the transmitter. In order to ascertain the stability of the spectral imaging system, the reference and background noise signals in both modes are repeatedly measured at least 10 times (1 ms for each measurement time), with a time lapse of at least 20 min between two consecutive measurements, and each measurement is performed after the THz TDS system undergoes a complete restart procedure. Moreover, the measurements have been repeated on several different days over the span of two weeks. A signal to noise ratio curve can be calculated through the reference and background noise signals for each measurement, and the average of signal to noise ratio curves for 10 measurements in both modes are shown in Figure S2. As can be seen from Figure S2, the signal to noise ratio in the through-transmission mode is better than 50 dB at the low-frequency end, and the effective measurement range is about 0~2.2 THz. In the reflection mode, it is better than 40 dB at the low-frequency end, and the effective measurement range is about 0~1.8 THz. In addition, from Figures S3 and S4, the reference signals in both modes are stable, even at very low frequency, and the standard deviation is rather small. The blue lines represent the mean of the reference signals measured for 10 times, and the length of the short black bars in the vertical direction represents the standard deviation. The red lines represent the mean of the noise signals measured for 10 times. Because the noise signals are much less than the reference signals, the red lines in Figures S3 and S4 are nearly straight lines around the zero. The inserts in Figures S3 and S4 show the enlarged drawings of the mean (the red lines) and standard deviation (the short black bars) of the noise signals. As can be seen from the enlarged drawings, the noise signals in both modes are relatively large from 0 to 0.05 THz, and they become very small after 0.05 THz. As the various defects to be considered in this paper are mainly detected at low frequency, the mean and standard deviation of the reference and noise signals are shown from 0 to 0.6 THz.

Because the best imaging frequency for the CFRP sample with a Teflon artificial insert defect is 0.075 THz, we pay special attention to the stability of the spectrum at 0.075 THz. From Figure S4 we can see that the reflected normalized reference power signal at 0.075 THz is very stable (0.3495 for the mean and 0.0072 for the standard deviation), and the standard deviation is much less than the normalized power change (0.0652) caused by the defect, which can be calculated from Figure 5 in the paper. In addition, the signal to noise ratio at 0.075 THz in the reflection mode is more than 28 dB, which again demonstrates the stability of the spectrum at 0.075 THz.

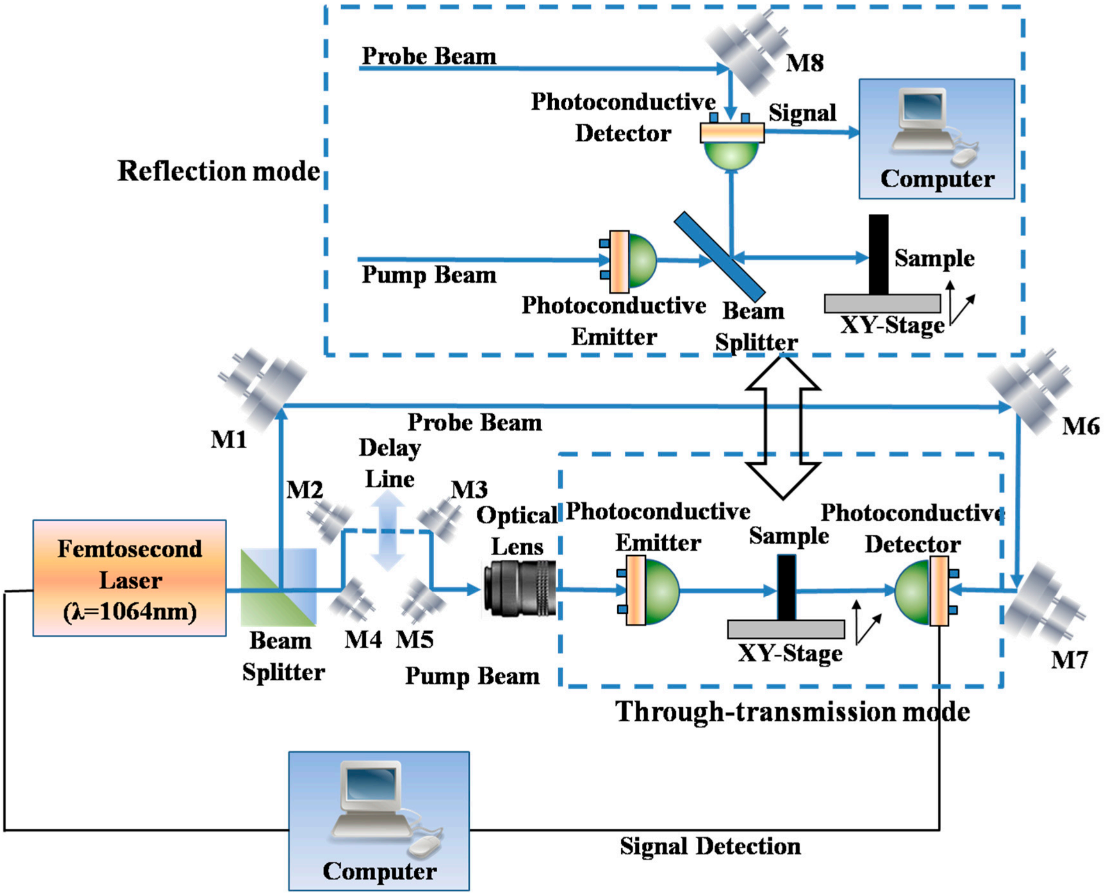

Figure S1. Schematic diagram of the THz TDS system (M1–M8: Mirrors).

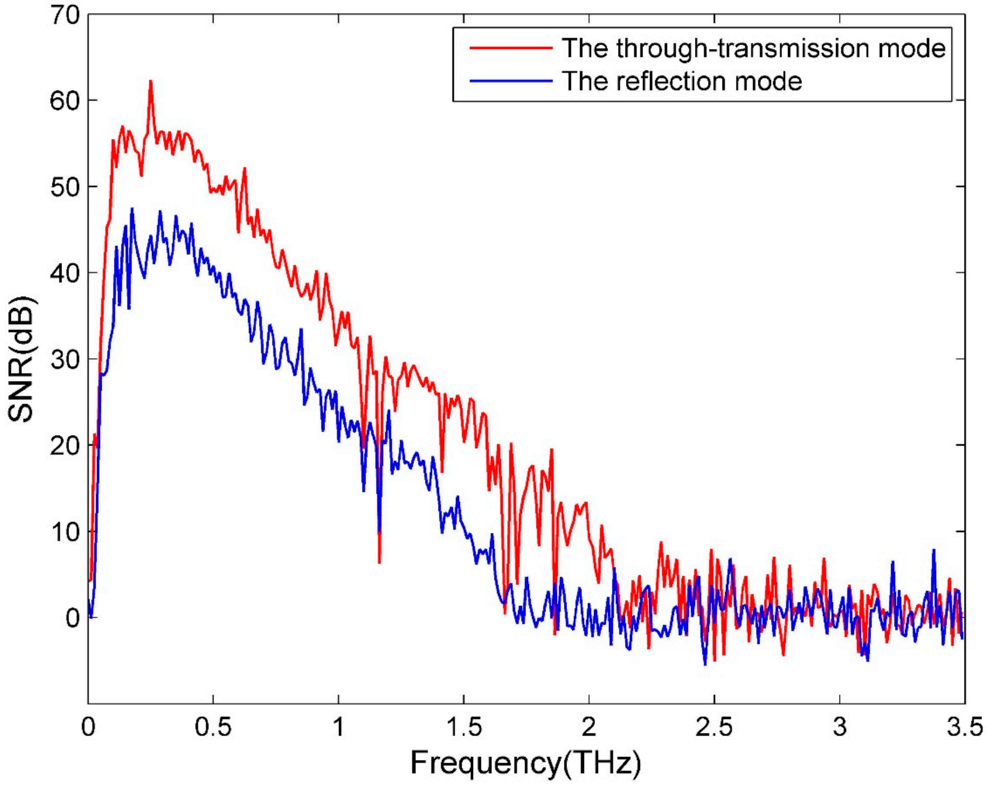

Figure S2. The signal to noise ratio of the THz TDS system in both the through-transmission and reflection mode.

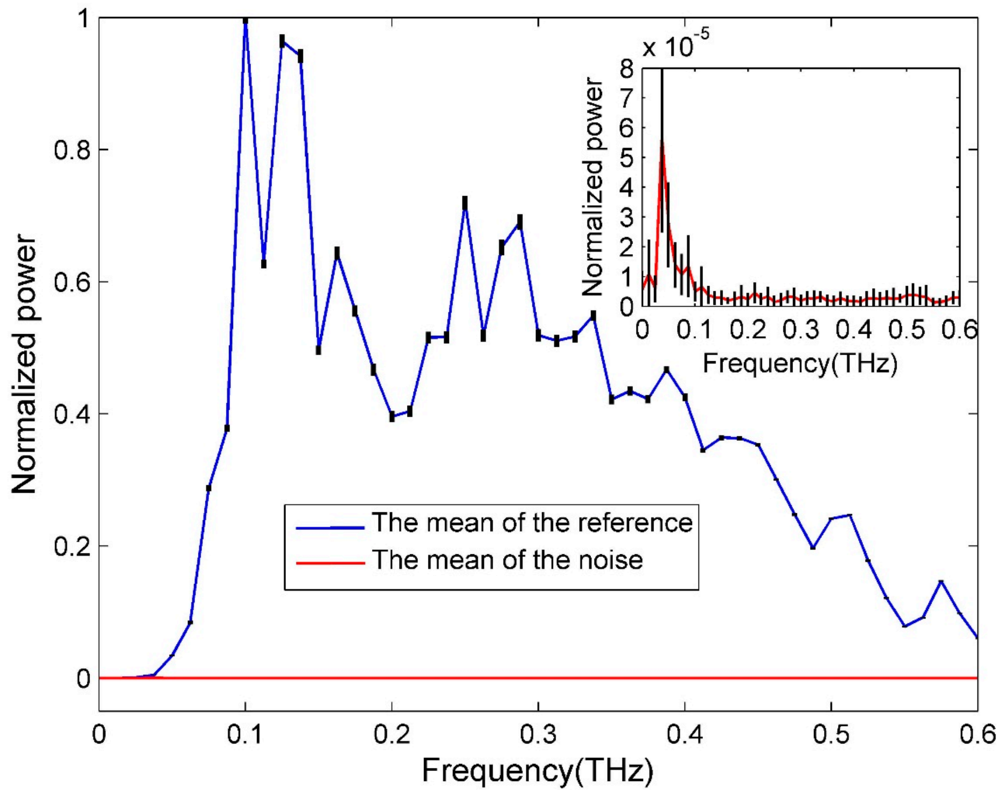

Figure S3. The corresponding mean and standard deviation of the reference and noise signals in the through-transmission mode.

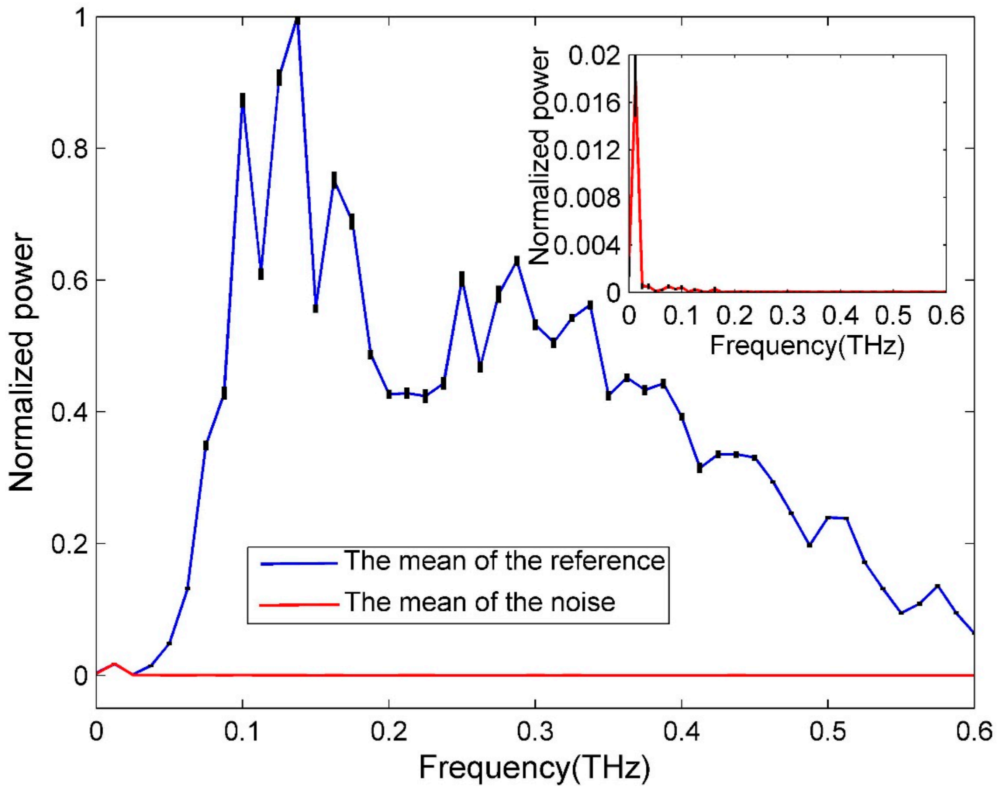

Figure S4. The corresponding mean and standard deviation of the reference and noise signals in the reflection mode.

2. The Significant Peaks for the Reference Signals

The significant peaks for the reference signals in both the through-transmission and the reflection mode are from the THz TDS system. As can be seen from Figures S3 and S4, the positions for the significant peaks in both modes are the same, and they are respectively at 0.1, 0.1375, 0.1625, 0.25, 0.2875, 0.3375, 0.3875, 0.45, 0.5 and 0.575 THz.

3. The Refractive Index of Epoxy Resin and Teflon

The frequency-dependent refractive index [1] in the through-transmission mode can be shown as:

$$n(\omega) = \frac{\varphi(\omega)c}{\omega d} + 1 \tag{1}$$

where $\varphi(\omega)$ is the phase difference between the reference and the sample, ω is the angular frequency, d is the sample thickness, and c is the THz wave propagation speed in the air.

Several epoxy resin and Teflon samples were fabricated separately, which have the same composition as those used in the CFRP sample with a Teflon artificial insert defect. They were measured in the through-transmission mode, and the refractive index was calculated by using Equation (1). As can be seen from Figure 6 in the paper, the maximum absolute difference of refractive index between epoxy resin and Teflon is at 0.075 THz. The inserts in Figure 6 show the refractive index of epoxy resin and Teflon respectively, and we can see that the maximum absolute difference at 0.075 THz mainly due to the epoxy resin. When THz wave is vertically incident, the Fabry–Pérot oscillation frequency period can be calculated as follows:

$$f = \frac{c}{2nd} \tag{2}$$

where n is the refractive index of the sample, and d is the thickness of the sample. Here, we use the mean refractive index of the epoxy resin sample (1.7289). The thickness of the sample is 1.564 mm, and it is difficult to fabricate an epoxy resin sample with the same thickness as those used in the defect samples. According to Equation (2), the calculated oscillation frequency period is about 0.0555. From the inserts in Figure 6, the measured oscillation frequencies is nearly 0.0563, 0.1187 and 0.1812 (marked with three asterisks), which have an agreement with the calculated oscillation frequency period. Hence, the strong peak at 0.075 THz is feasible, because the refractive index of epoxy resin and Teflon show no resonance features at this frequency.

4. Imaging Resolution

The lateral resolution of the THz TDS imaging system is usually determined by the diffraction-limited focal spot size [2]. Many complicated factors have an influence on the focal spot size, especially the detailed design of the optical system. On the whole, the focal spot size mainly depends on the wavelength and numerical aperture. The numerical aperture is given by the diameter of the lens and the focal length. The focal length of our system in the reflection mode is 75 mm, and the diameter of the lens is 38 mm. In other words, when the scanning step size is small enough, the lateral resolution of the images made using the frequency domain features depends on the wavelength, the diameter of the lens and the focal length. At the same time, the lateral resolution of the images made using the time domain features is intermediate between the high-frequency and low-frequency cases, because the features of the time domain pulse are a result of the addition of all the frequency components. Figure S6 shows the reflective imaging results of standard resolution test pieces with rectangular holes of different widths on an aluminum plate, when the scanning step size is 0.5 mm. The scale on the figures represents the width of the rectangular hole, which can be identified by the imaging results is the lateral resolution. As can be seen from Figure S6, the THz power images at the frequency from 1 to 0.075 THz becomes increasingly blurred, and the rectangular hole with 1 mm width cannot be seen completely in the THz power image at 0.075 THz. This is because the spot size of the THz beam increases with the decrease of frequency. The lateral

resolution of the peak-to-peak amplitude image in time domain is close to that of the THz power image at 0.5 THz. In addition, we can see that the lateral resolution of the THz power image at 0.075 THz is about 6 mm from the comparison between Figure S6e,f. Note that this is somewhat better than that given by the Rayleigh Criterion, which predicted a lateral resolution of 10 mm at 0.075 THz. In fact, we have found that the THz TDS system consistently better performs in terms of image resolution, as have been pointed out by others [3]. This is attributed to the Gaussian beam profile of the THz radiation, combined with the whisk broom scanning imaging mode, which resulted in improved image resolution.

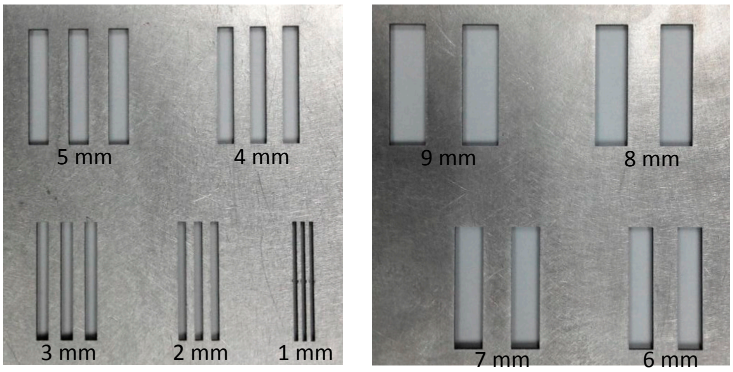

(**a**) (**b**)

Figure S5. Optical images of the standard resolution test pieces with rectangular holes of different widths on an aluminum plate: (**a**) 1–5 mm; (**b**) 6–9 mm.

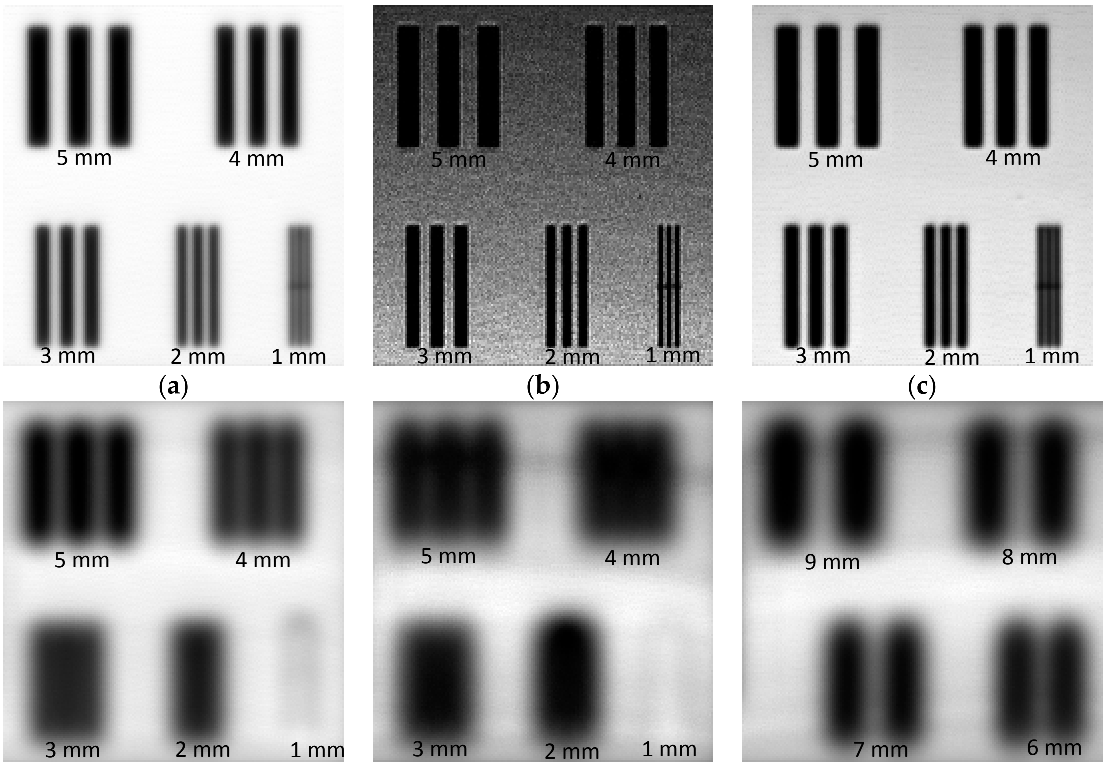

(**d**) (**e**) (**f**)

Figure S6. The reflective imaging results of standard resolution test pieces: (**a**) the peak-to-peak amplitude image in time domain; (**b**) THz power image at 1 THz; (**c**) THz power image at 0.5 THz; (**d**) THz power image at 0.1 THz; (**e**) THz power image at 0.075 THz for the rectangular holes of different widths from 1 to 5 mm; (**f**) THz power image at 0.075 THz for the rectangular holes of different widths from 6 to 9 mm.

References

1. Dorney, T.D.; Baraniuk, R.G.; Mittleman, D.M. Material parameter estimation with terahertz time-domain spectroscopy. *J. Opt. Soc. Am. A-Opt. Image Sci. Vis.* **2001**, *18*, 1562–1571.
2. Piston, D.W. Choosing objective lenses: the importance of numerical aperture and magnification in digital optical microscopy. *Biol. Bull.* **1998**, *195*, 1–4.
3. Taylor, Z.D.; Singh, R.S.; Brown, E.R.; Bjarnason, J.E.; Hanson, M.P.; Gossard, A.C. A reflection based, pulsed THz imaging system with 1 mm spatial resolution. In Proceedings of the IEEE/MTT-S International Microwave Symposium, Honolulu, HI, USA, 3–8 June 2007; pp. 1158–1161.
